# Inhibiting HDAC1 Enhances the Anti-Cancer Effects of Statins through Downregulation of GGTase-Iβ Expression

**DOI:** 10.3390/ijms18051010

**Published:** 2017-05-08

**Authors:** Ran Li, Ye-Hua Gan

**Affiliations:** 1 Central Laboratory, Peking University School and Hospital of Stomatology, 22 Zhongguancun Avenue South, Haidian District, Beijing 100081, China; lirankq@bjmu.edu.cn; 2 Department of Oral & Maxillofacial Surgery, Peking University School and Hospital of Stomatology, 22 Zhongguancun Avenue South, Haidian District, Beijing 100081, China

**Keywords:** head and neck cancers, statin, HDAC1, GGTase-Iβ, RhoA, chemotherapy

## Abstract

Hydroxy-methyl-glutaryl-coenzyme A (HMG-CoA) reductase inhibitors, namely statins, are potential anti-tumor agents. Previously, we showed that a pan-histone deacetylase (HDAC) inhibitor enhances the anti-tumor effects of the HMG-CoA inhibitor. However, the underlying mechanisms were not fully understood. Cancer cell lines (CAL-27 and SACC-83) were exposed to pan-HDAC inhibitor, or HDAC1 inhibitor, or geranylgeranyl transferase type I (GGTase-I) inhibitor alone or in combination with statin. Cell viability, apoptosis, migration, and invasion were assessed by Cell Count Kit-8, 4′,6-diamidino-2-phenylindole staining, and transwell assay, respectively. A xenograft model was used for assessing tumor growth in vivo. Western blot and real-time PCR were used to assess the expression of genes. We observed that inhibiting HDAC1 could enhance the anti-tumor effects of statins both in vitro and in vivo. Inhibiting HDAC1 blocked the statin-induced upregulation of geranylgeranyl transferase type Iβ subunit (GGTase-Iβ), resulting in an enhancement of the anti-cancer effects of statin. Overexpression of GGTase-Iβ or constitutively active RhoA abolished the enhancement by inhibiting HDAC1 on anti-tumor effects of statins. The HDAC1 inhibitor failed to enhance cytotoxicity in non-tumor primary cells treated with statin. Inhibiting HDAC1 enhanced the anti-cancer effects of statins through downregulation of GGTase-Iβ expression, and thus further inactivation of RhoA. A combination of statin with HDAC1 or GGTase-I inhibitor would be a new strategy for cancer chemotherapy.

## 1. Introduction

Inhibitors of 3-hydroxy-3-methylglutaryl coenzyme A (HMG-CoA) reductase, namely statins, are clinically used for lowering cholesterol by inhibiting the mevalonate pathway, with a relatively low price and good safety [1,2]. In addition to lowering cholesterol, statins also possess anti-cancer effects both in vitro and in vivo [3,4,5]. Although several mechanisms were proposed for the anti-tumor effects of statins, inhibition of geranylgeranylation is believed to be the major mechanism [6]. Geranylgeranylation is an important post-translational modification for many proteins (such as RhoA) to attach to the plasma membrane to function [7]. Statins inhibit the biosynthesis of geranylgeranyl pyrophosphate (GGPP), the substrate for geranylgeranylation, to induce anti-tumor effects [6,8]. Several clinical trials have indicated that intake of statins can reduce mortality and recurrence in patients with various kinds of cancer [9,10,11,12,13,14]. A combination of statins with first-line chemotherapeutic agents can improve the prognosis of cancer patients [15,16,17]. However, in a phase II trial, patients with advanced gastric adenocarcinoma showed no response to a high dose of lovastatin alone [18,19]. These studies suggest that statins, as potential anti-tumor drugs, may need to be used with other agents to enhance their own or others’ clinical effects on tumors. Therefore, it is still theoretically and clinically necessary to search for agents that can enhance the anti-tumor effects of statins or vice versa.

Geranylgeranyl transferase type I (GGTase-I) is a key enzyme for most geranylgeranylated proteins, such as Rho family of GTPases. GGTase-I consists of α and β subunits; the α subunit is shared with farnysl transferase, whereas the β subunit (GGTase-Iβ) determines the characteristics of GGTase-I [20]. Inhibitors of GGTase-I can induce anti-cancer effects both in vitro and in vivo [21,22]. Moreover, the conditional knockout of GGTase-Iβ subunit in mice can inhibit lung tumor formation [23,24]. Thus, GGTase-I is thought to be a potential target for cancer treatment [25].

Histone deacetylases (HDACs) control the de-acetylation of histones and non-histone proteins; classical HDACs contain members from HDAC1 to 11, which are essential for cell proliferation, differentiation, and apoptosis [26,27]. Aberrant expression or activation of HDACs is often found in various kinds of cancers, including breast, prostate, and gastric cancer [28,29,30]. Inhibitors for HDACs can induce anti-cancer effects both in vitro and in vivo [31,32]. Suberoylanilide hydroxamic acid (SAHA) is the first pan-HDAC inhibitor (inhibiting activity of all classical HDACs) approved by the U.S. Food and Drug Administration for the treatment of cutaneous T-cell lymphoma [33]. Several kinds of mechanisms are involved in the anti-cancer effects of HDAC inhibitors [34]. Induction of reactive oxide species (ROS), activation of death receptor, or PTEN are involved in HDAC inhibitor-induced anti-cancer effects [35,36,37,38]. Our group previously observed that the pan-HDAC inhibitor trichostatin A (TSA) synergistically induced apoptosis with HMG-CoA inhibitor, mevastatin, in HeLa cells [39]. However, the underlying mechanisms remain to be fully understood. Recently, more attention has been focused on the combination of HDAC inhibitors with other anti-cancer agents [34,40]. The development of new combinations of anti-cancer agents could be an important strategy for fighting cancer chemoresistance.

In this study, we show that, similar to the pan-HDAC inhibitor, inhibition of HDAC1 resulted in a significant enhancement in the anti-cancer effects of statins both in vitro and in vivo, and that down-regulation of GGTase-Iβ expression by the inhibition of HDAC1, resulting in inactivation of RhoA, was responsible for this enhancement.

## 2. Results

### 2.1. Suberoylanilide Hydroxamic Acid (SAHA) Enhanced Statin-Induced Anti-Cancer Effects

**Figure 1:**
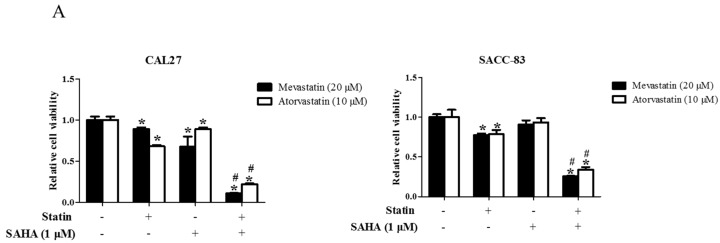

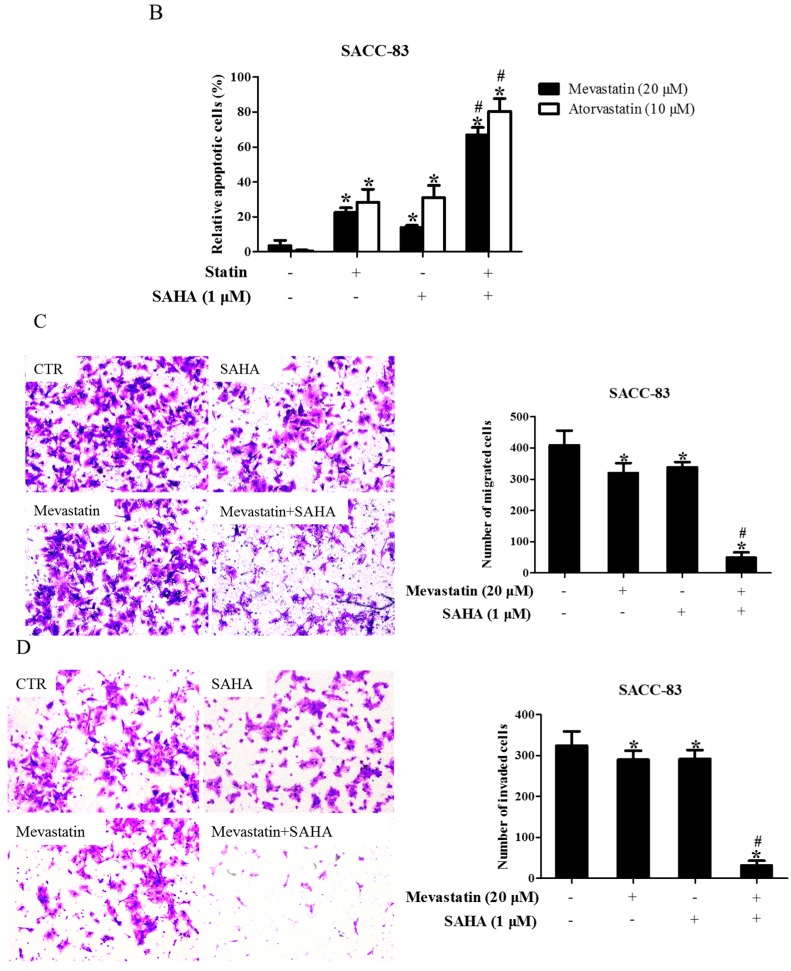
Suberoylanilide Hydroxamic Acid (SAHA) enhanced statin-induced anti-cancer effects. SACC-83 and CAL27 cells were exposed to either statin (mevastatin/atorvastatin), SAHA, or both for 48 h. Cell viability was assessed by Cell Count Kit-8 assay, *n* = 4 (**A**); The rate of apoptotic SACC-83 cells was quantified, *n* = 6 (**B**); Microphotographs of cell migration (**C**) or invasion (**D**) of cells after different treatment for 16 h, *n* = 6 (20×). * *P* < 0.05 vs. control group; # *P* < 0.05 vs. SAHA or statin (mevastatin/atorvastatin) group.

Pan-HDAC inhibitor SAHA significantly enhanced the mevastatin- or atorvastatin-induced apoptosis and inhibition of cell proliferation in both CAL27 and SACC-83 cells (Figure 1A,B), whereas SAHA or mevastatin or atorvastatin alone only slightly inhibited cell proliferation. Moreover, SAHA also significantly promoted statin-induced inhibition of transwell migration (Figure 1C) and invasion (Figure 1D) in SACC-83 cells.

### 2.2. Inhibition of HDAC1 Was Responsible for Pan-HDAC Inhibitor to Enhance Anti-Cancer Effects of Statins

**Figure 2:**
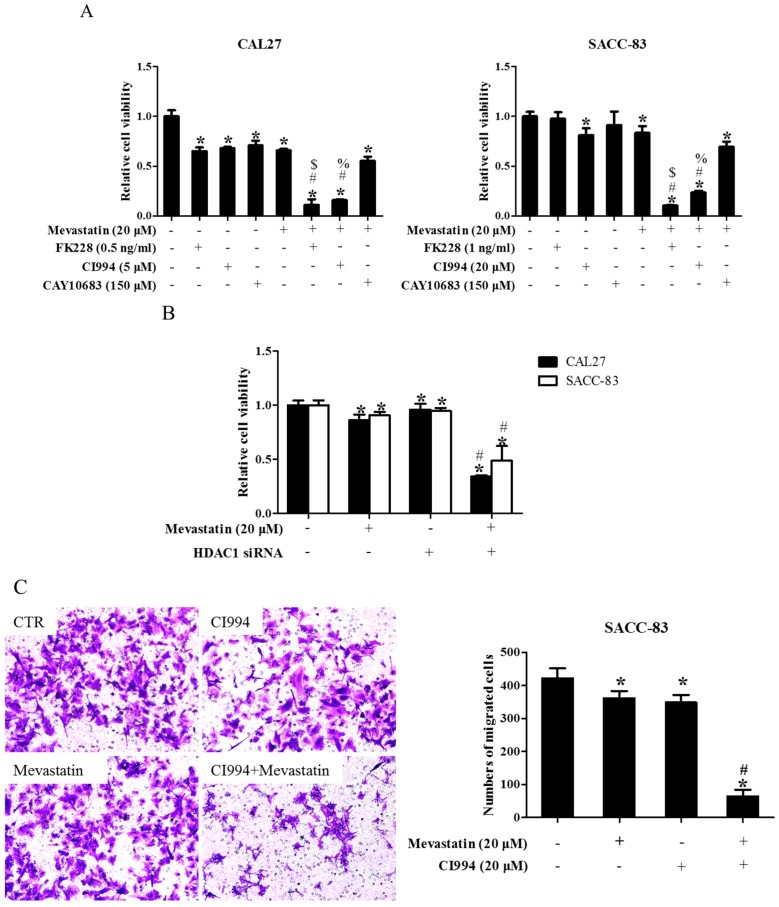

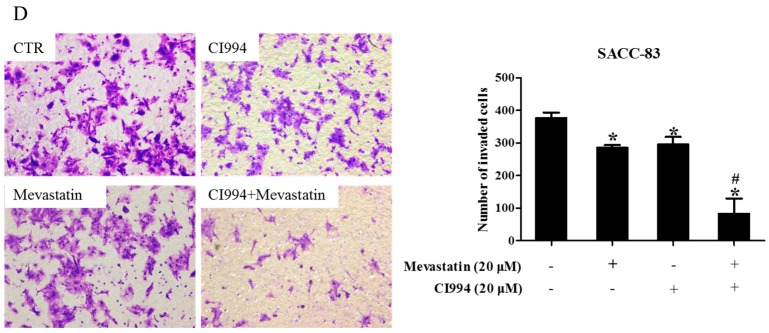
Inhibition of HDAC1 also enhanced statin-induced anti-cancer effects. (**A**) SACC-83 and CAL-27 cells were either exposed to various kinds of HDAC inhibitors (FK228, inhibitor for HDAC1&2; CI994, inhibitor for HDAC1; CAY10683, inhibitor for HDAC2), or together with mevastatin for 48 h. Cell viability assessed by CCK8 assay. * *P* < 0.05 vs. the control group; # *P* < 0.05 vs. mevastatin group; $ *P* < 0.05 vs. FK228 group; % *P* < 0.05 vs. CI994 group, *n* = 4; (**B**) SACC-83 and CAL27 cells were treated with either HDAC1 siRNA or mevastatin, or both. Cell viability was assessed by CCK8 after 48 h. * *P* < 0.05 vs. the control group; # *P* < 0.05 vs. HDAC1 siRNA or mevastatin group, *n* = 4; Microphotographs of cell migration (**C**) and invasion (**D**) in SACC-83 cells after 16 h treatment with CI994 and mevastatin (20×). * *P* < 0.05 vs. the control group; # *P* < 0.05 vs. CI994 or mevastatin group, *n* = 6.

To narrow down which HDAC was involved in the enhancement of statin-induced anti-cancer effects, CAL27 and SACC-83 cells were exposed to various HDAC inhibitors in the presence of mevastatin. Inhibition of HDAC1, 2, 3, 6, 8, and 10 by PCI24781 could enhance the mevastatin-induced inhibition of cell proliferation, whereas inhibiting HDAC3, 6, and 8 by RGFP966, tubacin, and PCI34051, respectively, or HDAC4, 5, 7, and 9 by MC1568, and knocking down HDAC10 and 11 by siRNAs, failed to do so (Figure S1), suggesting that HDAC1 or HDAC2 or both was involved in the pan-HDAC inhibitor-induced enhancement of the anti-cancer effects of mevastatin. HDAC10 and HDAC11 were successfully knocked down by siRNAs (Figures S2 and S3). We further narrowed it down to HDAC1, as shown in Figure 2A: FK228 (an inhibitor of HDAC1&2) and CI994 (an inhibitor of HDAC1) could both enhance the mevastatin-induced inhibition of proliferation of CAL27 or SACC-83 cells, whereas CAY10683 (an inhibitor of HDAC2) failed to do so. Similar results were observed in CAL27 cells treated with FK228 or CI994 or CAY10683 in the presence of atorvastatin (Figure S4). Moreover, knockdown of HDAC1 could also significantly enhance the mevastatin-induced inhibition of proliferation of the two cell lines examined (Figure 2B). HDAC1, but not HDAC2, 3, and 8, was knocked down by HDAC1 siRNA (Figures S5 and S6). In addition, GGPP abolished the enhancement of statin-induced inhibition of cell proliferation by CI994 (Figure S7), suggesting that geranylgeranylation was critical for this enhancement. Inhibition of HDAC1 by CI994 also promoted mevastatin-induced inhibition of transwell migration (Figure 2C) and invasion (Figure 2D) in SACC-83 cells. These data showed that pan-HDAC inhibitor SAHA enhanced the anti-cancer effects of mevastatin or atorvastatin through inhibition of HDAC1.

### 2.3. HDAC1 Inhibitor and Atorvastatin Synergistically Inhibited CAL27 Xenograft Growth in Nude Mice

**Figure 3:**
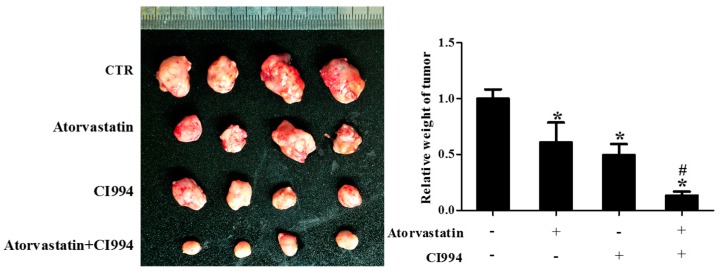
HDAC1 inhibitor and atorvastatin synergistically inhibited CAL27 xenograft growth in nude mice. Photographs and weights of xenograft tumors. Nude mice were inoculated with CAL27 cells, and treated with either atorvastatin or CI994, or both, for three weeks. * *P* < 0.05 vs. control group; # *P* < 0.05 vs. atorvastatin or CI994 group, *n* = 4.

As shown in Figure 3, the weight of CAL27 xenografts in the group received combinational treatment with CI994 and atorvastatin was significantly lower than that of the groups that received non-treatment, atorvastatin, or CI994.

### 2.4. Inhibition of GGTase-Iβ also Enhanced the Anti-Cancer Effects of Statin

**Figure 4:**
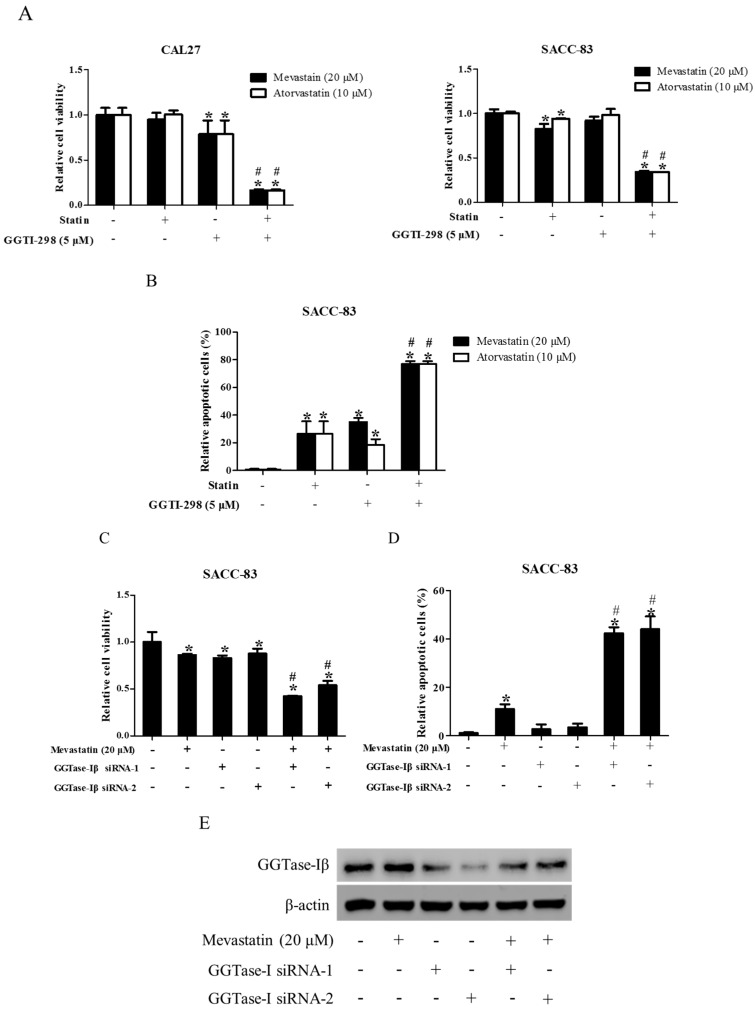
Inhibition of GGTase-Iβ also enhanced statin-induced anti-cancer effects. SACC-83 and CAL27 cells were exposed to either statin (mevastatin/atorvastatin), or GGTI-298, or both 48 h. Cell viability was assessed by CCK8 assay, *n* = 4 (**A**); Rate of cell apoptosis was quantified by DAPI staining, *n* = 6 (**B**); * *P* < 0.05 vs. the control group; # *P* < 0.05 vs. the statin (mevastatin or atorvastatin) group or GGTI-298 group. SACC-83 cells were respectively transfected with GGTase-Iβ siRNA-1 or -2, and co-treated with mevastatin or not for 48 h. Cell viability was assessed by CCK8, *n* = 4 (**C**); Rate of cell apoptosis was quantified by DAPI staining (**D**); * *P* < 0.05 vs. control group; # *P* < 0.05 vs. mevastatin or GGTase-Iβ siRNA group, *n* = 6; (**E**) Protein expression of GGTase-Iβ was assessed by Western blot after treatment with mevastatin, GGTase-Iβ siRNA, or both.

In previous study, we speculated that downregulation of GGTase-Iβ by pan-HDAC inhibitor TSA might contribute to the TSA enhancement of statin-induced apoptosis and inhibition of proliferation [39]. To confirm this speculation, we first examined whether the inhibition of GGTase-I could generate similar effects to SAHA on the anti-cancer effects of statins in CAL27 and SACC-83 cells. As shown in Figure 4, co-treatment of mevastatin/atorvastatin and GGTase-I inhibitor GGTI-298 synergistically inhibited cell proliferation (Figure 4A) and induced apoptosis (Figure 4B), whereas GGTI-298 alone only slightly inhibited the proliferation of CAL27 cells, but did not affect SACC-83 cells. Moreover, knockdown of GGTase-Iβ by siRNAs also enhanced the statin-induced inhibition of cell proliferation (Figure 4C) and apoptosis (Figure 4D).

### 2.5. GGTase-Iβ Mediated the Synergistic Anti-Cancer Effects of HDAC1 Inhibitor and Statin

**Figure 5:**
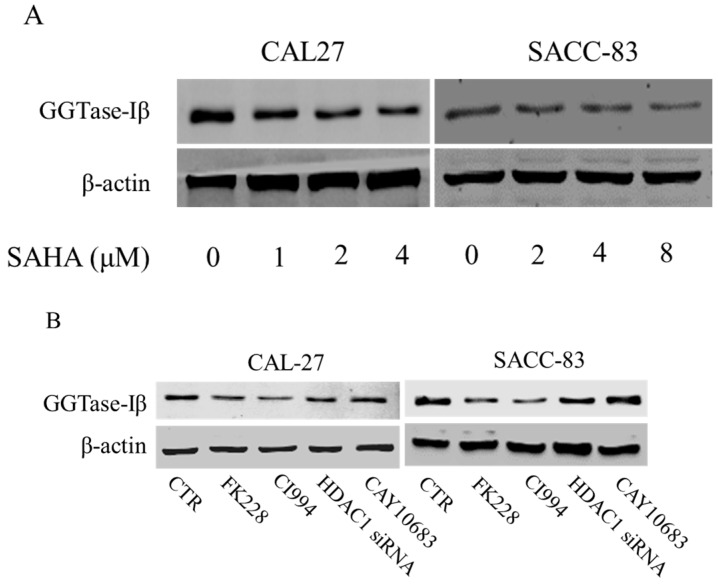

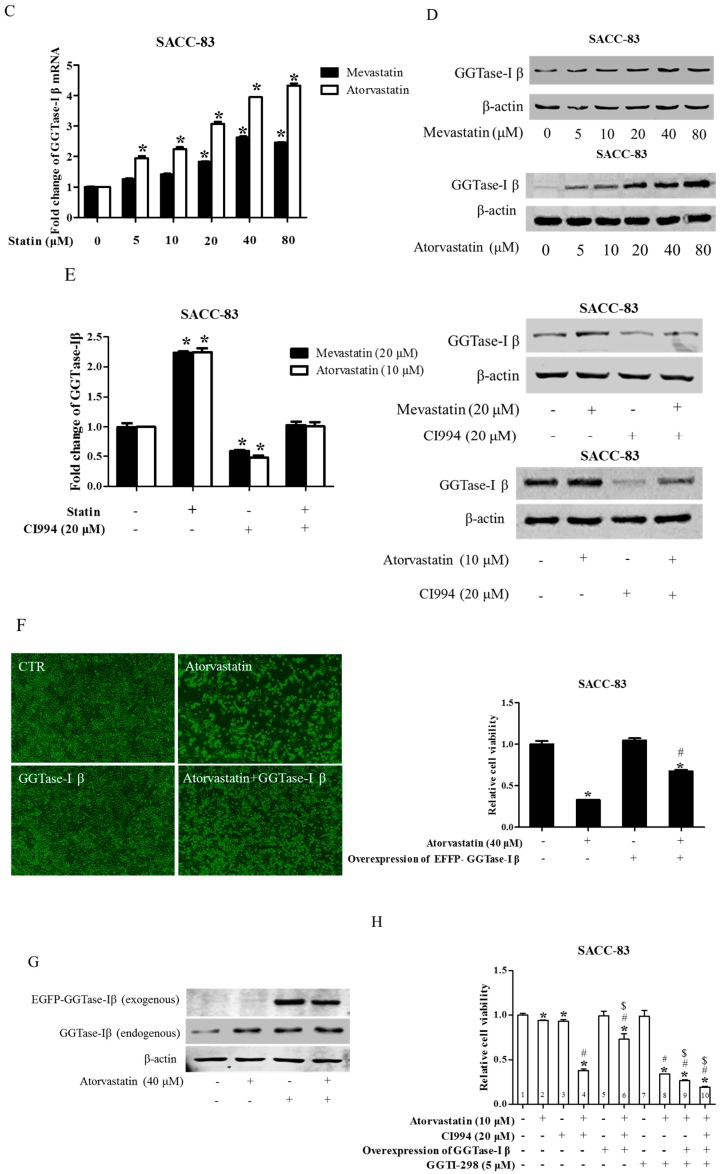

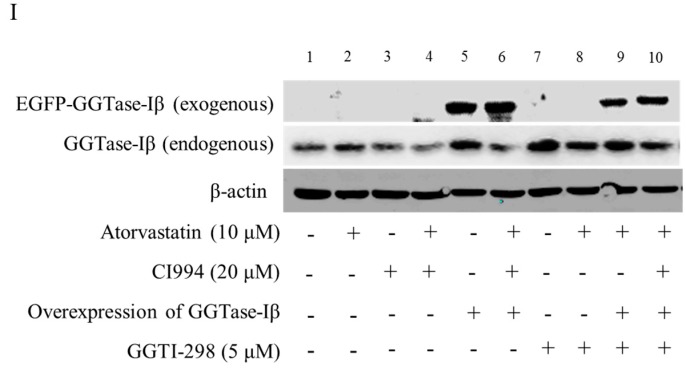
GGTase-Iβ mediated the synergistic anti-cancer effects of HDAC1 inhibitor and statin. SACC-83 and CAL27 cells were treated with either different doses of SAHA (**A**) or various kinds of HDAC inhibitor/siRNA (**B**); after 30 h treatment the total protein was extracted and subjected to Western blot. SACC-83 cells were exposed to different doses of statins (mevastatin/atorvastatin); after 30 h treatment, total mRNA and protein were extracted and subjected to either real-time PCR (**C**) or Western blot (**D**) for analysis; (**E**) GGTase-Iβ protein expression after treatment with either statin (mevastatin/atorvastatin), or CI994, or both for 30 h in SACC-83 cells; (**F**) SACC-83 cells were transfected with either EGFP-fused GGTase-Iβ, or an empty vector, after transfection cells were exposed to atorvastatin at a concentration of 40 μM for 48 h. Cell viability was assessed by CCK8; (**G**) Total protein was also extracted and subjected to analysis by Western blot. * *P* < 0.05 vs. the control group; # *P* < 0.05 vs. atorvastatin or GGTase-Iβ group, *n* = 4. SACC-83 cells were transfected with either an empty vector (pEGFP-C1) or EGFP-fused GGTase-Iβ, and then exposed to different kinds of treatment. Cell viability was assessed by CCK8 assay after 48 h treatment (**H**); Total protein was extracted and subjected to Western blot (**I**). * *P* < 0.05 vs. the control group; # *P* < 0.05 vs. atorvastatin group; $ *P* < 0.05 vs. combination of atorvastatin and CI994 group, *n* = 4.

We next examined whether GGTase-Iβ mediated the enhancement by pan-HDAC inhibitor or HDAC1 inhibitor of anti-cancer effects of statins in cells. SAHA inhibited protein expression of GGTase-Iβ in a dose-dependent manner (Figure 5A); GGTase-Iβ expression was also downregulated by treatment with FK228, CI994, or HDAC1 siRNA, but not by an inhibitor of HDAC2, CAY10683 (Figure 5B). Moreover, inhibition of HDAC1 by CI994 could slightly downregulate membrane translocation (activation) of RhoA without affecting its protein expression (Figure S8). Interestingly, knockdown of HDAC1 did not influence GGTase-Iβ promoter activity by luciferase assay (Figure S9). However, inhibition of new protein synthesis by cycloheximide (CHX) dramatically induced expression of GGTase-Iβ mRNA, and rescued CI994-induced downregulation of GGTase-Iβ (Figure S10). Mevastatin and atorvastatin both upregulated levels of GGTase-Iβ mRNA (Figure 5C) and protein (Figure 5D). Treatment with CI994 blocked statin-induced upregulation of GGTase-Iβ mRNA (Figure S11) and protein (Figure 5E). Moreover, overexpression of GGTase-Iβ (fused with EGFP) partially reversed atorvastatin- or mevastatin-induced inhibition of cell proliferation, whereas overexpression of GGTase-Iβ (fused with EGFP) alone did not influence cell viability (Figure 5F and Figure S12). As shown in Figure 5G, overexpression of GGTase-Iβ (fused with EGFP) also unexpectedly upregulated endogenous GGTase-Iβ protein expression, similarly to atorvastatin; a combination of atorvastatin and overexpression of GGTase-Iβ failed to induce endogenous GGTase-Iβ protein expression further than each alone. Moreover, as shown in Figure 5H, overexpression of GGTase-Iβ completely abolished CI994 enhancement of atorvastatin-induced inhibition of cell proliferation (columns 1–6); GGTase-Iβ inhibitor GGTI-298 also enhanced atorvastatin-induced inhibition of cell proliferation (columns 2, 7, and 8), similarly to CI994 (column 4), whereas GGTI-298 alone did not affect cell proliferation (column 7); GGTI-298 also reversed the abolishing effect of exogenous GGTase-Iβ on CI994 enhancement of atorvastatin-induced inhibition of cell proliferation (columns 6 and 10); exogenous GGTase-Iβ failed to affect GGTI-298 enhancement of atorvastatin-induced inhibition of cell proliferation (columns 8 and 9). As shown in Figure 5I, GGTase-Iβ protein was correspondingly changed by the treatments indicated; GGTI-298 also upregulated endogenous GGTase-Iβ expression, similarly to atorvastatin and exogenous GGTase-Iβ; CI994 completely blocked atorvastatin- or exogenous GGTase-Iβ-induced upregulation of endogenous GGTase-Iβ expression (lanes 3, 4, and 6), but failed to block GGTI-298-induced upregulation of endogenous GGTase-Iβ expression (lane 10).

### 2.6. Overexpression of Constitutively Active RhoA Abolished Enhancement by HDAC1 or GGTase-I Inhibitor of Statin-Induced Anti-Cancer Effects

**Figure 6:**
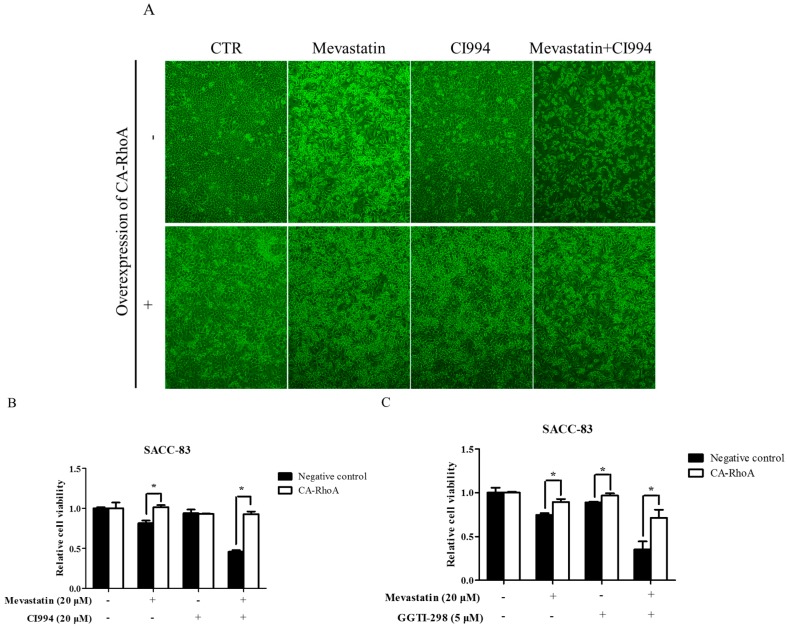
Overexpression of constitutively active RhoA abolished enhancement by HDAC1 or GGTase-I inhibitor of statin-induced anti-cancer effects. SACC-83 cells stably transfected with constitutively active RhoA or not were exposed to either mevastatin, or CI994, or both for 48 h. (**A**) Microphotographs after treatment (10×); (**B**) cell viability assessed by CCK8 assay; (**C**) SACC-83 cells stable transfected with constitutively active RhoA or not were exposed to either mevastatin, GGTI-298, or both for 48 h. Cell viability was assessed by CCK8 assay. * *P* < 0.05, *n* = 4.

To further explore the downstream effector for the combination of HDAC1 inhibitor and statins, EGFP fused constitutively active RhoA (RhoA Q63L, CA-RhoA) was stably transfected in SACC-83 cells [41]. Western blot confirmed the overexpression of CA-RhoA in SACC-83 cells (Figure S13). As shown in Figure 6A,B, overexpression of CA-RhoA rescued mevastatin-induced inhibition of cell proliferation, but not affect CI994-induced inhibition of cell viability, whereas overexpression of CA-RhoA abolished the enhancement by HDAC1 inhibitor of statin-induced cell proliferation inhibition and apoptosis. Moreover, overexpression of CA-RhoA also abolished the enhancement of mevastatin-induced inhibition of cell proliferation in SACC-83 cells (Figure 6C). 

### 2.7. HDAC1 Inhibitor Did Not Enhance Statin-Induced Inhibition of Cell Proliferation in Non-Tumor Primary Cells

**Figure 7:**
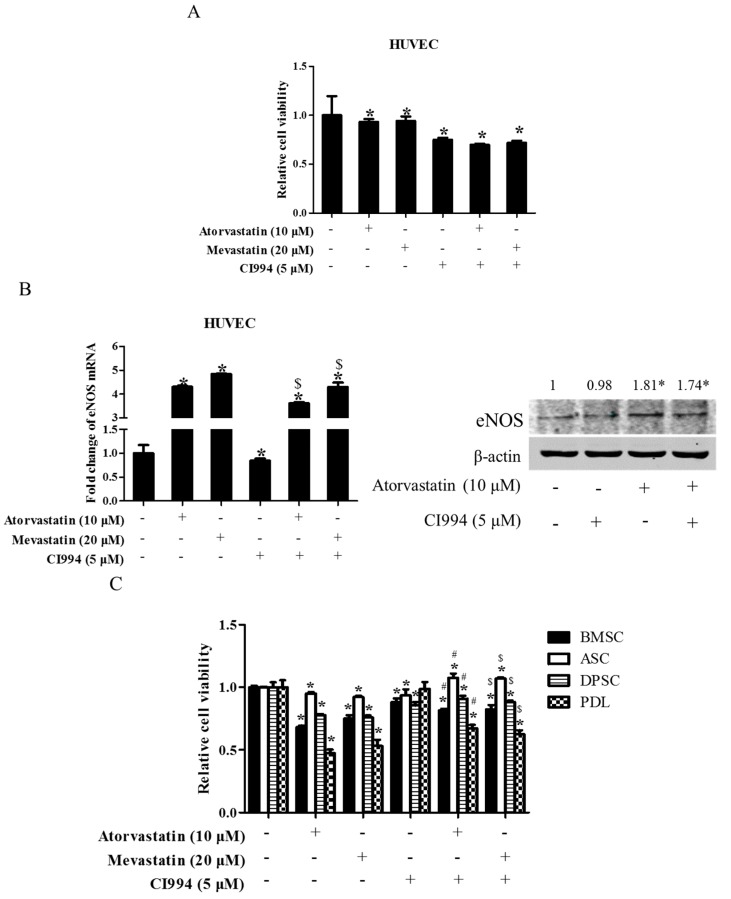
HDAC1 inhibitor did not enhance statin-induced inhibition of cell proliferation in non-tumor primary cells. HUVEC cells were exposed to either statin (mevastatin 20 μM/atorvastatin 10 μM), or CI994 (5 μM), or both 48 h. Cell viability was assessed by a CCK8 assay (**A**); Expression of eNOS was analyzed by real-time PCR and Western blot (**B**); * *P* < 0.05 vs. the control group; $ *P* < 0.05 vs. CI994 group, *n* = 3. Non-tumor primary cells (BMSC, DPSC, PDL, ASC) were exposed to either statin (mevastatin 20 μM/atorvastatin 10 μM), or CI994 (5 μM), or both for 48 h. Cell viability assessed by CCK8 assay (**C**); * *P* < 0.05 vs. the control group; # *P* < 0.05 vs. mevastatin group; $ *P* < 0.05 vs. atorvastatin group, *n* = 4.

To test whether or not the combination of statin and CI994 could also induce synergistic cytotoxicity in non-tumor primary cells, several kinds of human-derived primary cells were exposed to statin, CI994, or both for 48 h. As shown in Figure 7A, treatment with atorvastatin, mevastatin, and CI994 alone all slightly inhibited cell proliferation in HUVEC cells, whereas combinational treatment with statin and CI994 did not induce further inhibition of cell proliferation. Interestingly, atorvastatin or combinational treatment with statin and CI994 resulted in upregulation of endothelial nitric oxide synthase (eNOS) expression, whereas CI994 slightly downregulated eNOS expression (Figure 7B). Similarly, CI994 did not enhance atorvastatin- /mevastatin-induced inhibition of cell proliferation in other non-tumor primary cells, including BMSC, PDL, DPSC, and ASC (Figure 7C).

## 3. Discussion

In the present study, we present evidence that the inhibition of HDAC1 enhances the anti-cancer effects of statins. Inhibition of HDAC1 could enhance statin-induced inhibition of cell proliferation, apoptosis, migration, and invasion in two cancer cell lines. The enhancement by inhibiting HDAC1 of anti-cancer effects of statins was similar to that of pan-HDAC inhibitor SAHA. Although we previously also observed this enhancement using a pan-HDAC inhibitor, we did not identify which HDAC was responsible for it [39]. In the present study, we narrowed it down to HDAC1 by applying its specific inhibitor and siRNAs, and also excluded the involvement of other HDACs by inhibitors or siRNAs in the assays of cell proliferation. Therefore, the inhibition of HDAC1 was responsible for the pan-HDAC inhibitor-induced enhancement of statin anti-cancer effects. The enhancement by inhibiting HDAC1 of statin-induced anti-cancer effects was also confirmed in nude mice with xenografts. Considering that a strong expression of HDAC1 was correlated with poor prognosis in cancer patients [42,43], and that HDAC1 is also overexpressed in cancer stem cells and modulates their function [44], the inhibition of HDAC1 could be an important strategy in cancer treatment. Given that a combination of statin and HDAC1 inhibitor produced a synergistic anti-cancer effect, this combination could be a potential new regimen in cancer chemotherapy.

Downregulation of GGTase-Iβ expression by inhibition of HDAC1 was the underlying mechanism for HDAC1 enhancement of statin-induced anticancer effects. The pan-HDAC inhibitor SAHA or HDAC1 inhibitor CI994 or siRNAs could all downregulate GGTase-Iβ. Moreover, inhibition of GGTase-I by a specific inhibitor or siRNAs also enhanced statin-induced apoptosis and inhibition of proliferation. In most cases, HDAC inhibitors promote the expression of genes by loosening the structure of chromatin [45,46]. However, inhibition of HDAC1 could inhibit the expression of GGTase-Iβ. Our results showed that inhibition of GGTase-Iβ expression by inhibiting HDAC1 was not due to the influence on its promoter activity, but to the destabilization of its mRNA by some proteins regulated by HDAC1, for which detailed mechanisms need to be further explored.

Interestingly, statins induced GGTase-Iβ expression while they inhibited cell proliferation. This phenomenon might be due to the negative feedback mechanism, i.e., the depletion of GGPP by statins somehow trigged the response by upregulating GGTase-Iβ expression to boost transferring GGPP to the target proteins in order to compensate for the decrease in geranylgeranylation due to the depletion of its substrate GGPP. This explanation could be supported by the results that overexpression of GGTase-Iβ could partially rescue statin-induced inhibition of cell proliferation. However, pan-HDAC inhibitor or inhibition of HDAC1 downregulated GGTase-Iβ expression and, moreover, blocked statin-induced upregulation of GGTase-Iβ expression. This blockade of statin-induced upregulation of GGTase-Iβ expression prevented the rescue response due to the negative feedback mechanism within the cells, leading to geranylgeranylation being more difficult. The blockade of statin-induced upregulation of GGTase-Iβ expression was further proven to be responsible for HDAC1 inhibitor enhancement of statin-induced anti-cancer effects, as the overexpression of exogenous GGTase-Iβ completely abolished this enhancement; in the presence of GGTase-I inhibitor GGTI-298, overexpression of GGTase-Iβ failed to abolish this enhancement. Therefore, a combination of statin and GGTase-I inhibitor or agent inhibiting GGTase-Iβ expression could also be a potential new regimen in cancer chemotherapy.

In addition, inhibition of GGTase-Iβ by GGTI-298 also resulted in upregulation of endogenous GGTase-Iβ expression. This was also likely due to the negative feedback mechanism, again demonstrating the importance of geranylgeranylation in homeostasis. What we did expect, however, was that overexpression of exogenous GGTase-Iβ also promoted endogenous GGTase-Iβ expression. It seemed that GGTase-Iβ might also be regulated by the positive feedback mechanism, i.e., the more GGTase-Iβ exists the more GGTase-Iβ is induced to some extent. However, overexpression of exogenous GGTase-Iβ did not affect the proliferation of the cells examined. It appeared that the exogenous GGTase-Iβ and statin might both be dependent on the HDAC1 signaling pathway in the upregulation of GGTase-Iβ expression, for inhibition of HDAC1 could completely block either exogenous GGTase-Iβ-induced or statin-induced upregulation of GGTase-Iβ expression (Figure 4I). Whereas GGTase-Iβ inhibitor GGTI-298 appeared not to be dependent on the HDAC1 signaling pathway, inhibition of HDAC1 failed to block the GGTI-298-induced upregulation of GGTase-Iβ expression (Figure 4I). It should also be noted that the endogenous GGTase-Iβ protein expression was not further upregulated by the combinational treatment with the statin and overexpression of exogenous GGTase-Iβ compared to each alone, implying that the upregulation of GGTase-Iβ might be limited to some maximum level by unknown mechanisms, or one factor such as overexpression of exogenous GGTase-Iβ overwhelmed the other such as atorvastatin in upregulating GGTase-Iβ expression through the same pathway, e.g., the HDAC1 signaling pathway.

RhoA was determined as the key target for the enhancement of statin-induced anti-cancer effects by HDAC1 inhibitor. In our previous study, we did not provide sufficient data to prove that RhoA is a key target of the combination of mevastatin and TSA, although we observed that mevastatin greatly, and TSA slightly, inhibits membrane translocation of RhoA, and the combination of TSA and mevastatin synergistically depletes membrane-bound RhoA [39]. In the current study, inhibition of HDAC1 also slightly inhibited membrane translocation of RhoA (Figure S8). Therefore, we did rescue assay with constitutively active RhoA (CA-RhoA). Stable transfection of CA-RhoA, not only rescued mevastatin-induced inhibition of cell viability, but also abolished the enhancement by inhibiting HDAC1 of mevastatin-induced cell proliferation inhibition and apoptosis. These results strongly suggested that RhoA was the key target among the 22 members of the Rho family for cell survival and proliferation, underlying the statin treatment and the combinational treatment of statin and HDAC1 inhibitor. The abolishment by overexpression of CA-RhoA further confirmed that the enhancement of statin-induced inhibition of cell proliferation was due to further inhibition of geranylgeranylation by inhibiting GGTase-Iβ, but not inhibition of HDAC1, although it was reported that statins could inhibit HDAC activity as the HDAC inhibitor did [47]. In contrast to cancer cells, HDAC1 inhibitor CI994 did not show any enhancement of statin-induced inhibition of proliferation in several primary cells, including HUVEC (related to the cardiovascular system), BMSC (related to hematopoiesis and the immune system), PDL, DPSC, and ASC. In particular, the combination of CI994 and atorvastatin in HUVEC resulted in an increase in eNOS, which benefits the cardiovascular system. Therefore, the combination of HDAC1 inhibitor and statin might have clinical advantages, i.e., the combination would possibly produce fewer adverse effects on the cardiovascular system, hematopoiesis, and the immune system. The reason why CI994 did not enhance statin-induced proliferation in non-cancer cells remains unknown. It is likely that the proliferation of cancer cells was more dependent on geranylgeranylated proteins such as RhoA, Rac1, etc., leading to greater sensitivity to the depletion of GGPP and inhibition of GGTase-Iβ expression than in the non-cancer cells.

In the current study, only mevastatin and atorvastatin were used with a pan-HDAC inhibitor or the HDAC1 inhibitor to examine anti-cancer effects in two cell lines. The two statins are lipophilic. We believe that other lipophilic statins such as simvastatin, in combination with the HDAC1 inhibitor, could produce similar results to those of mevastatin and atorvastatin in the two cell lines. However, we are less confident that the non-lipophilic statins such as rosuvastatin and pravastatin would produce similar results in the two cell lines, since the non-lipophilic statins show liver-specific activity [48,49,50]. Therefore, for the treatment of non-liver cancers, lipophilic statins may be better than hydrophilic statins, or vice versa. Future studies are needed to address these points.

In conclusion, we showed that the inhibition of HDAC1 was responsible for pan-HDAC inhibitors enhancing the anti-cancer effects of statins; the inhibition of HDAC1 enhanced statin-induced inhibition of proliferation through a downregulation of GGTase-Iβ expression in cancer cells, but not in non-cancer primary cells. Our results suggested that the combination of HDAC1 inhibitor and a statin would be a potential new regimen for cancer therapeutics.

## 4. Materials and Methods

### 4.1. Cell Lines

Human tongue squamous carcinoma-derived CAL-27 cells were incubated in Dulbecco’s modified Eagle’s medium (GIBCO, Grand Island, NY, USA) with 10% fetal bovine serum at 37 °C under 5% CO_2_ [51]; human salivary adenoid cystic cancer (SACC)-derived SACC-83 cells were incubated in RPMI medium 1640 (GIBCO) with 10% FBS at 37 °C with 5% CO_2_ [52]. Human bone mesenchymal stem cells (BMSC) and periodontal ligament (PDL) stem cells were cultured as previously described [53,54]; human dental pulp stem cells (DPSC) and adipose-derived stem cells (ASC) were incubated in Dulbecco’s modified Eagle’s medium (GIBCO) with 10% fetal bovine serum at 37 °C under 5% CO_2_. Human umbilical vein endothelial cells (HUVECs) were cultured at 37 °C with 5% CO_2_ in medium 200 with low serum growth supplement (LSGS) kit (GIBCO).

### 4.2. Reagents and Antibodies

SAHA, FK228, CI994, CAY10481, PCI24781, PCI34051, and RGFP966 were purchased from Selleck Chemicals (Houston, TX, USA). GGTase-I specific inhibitor GGTI-298 was purchased from Sigma-Aldrich (St. Louis, MO, USA). Anti-human GGTase-Iβ subunit and RhoA antibodies were purchased from Santa Cruz Biotechnology (Santa Cruz, CA, USA).

### 4.3. Plasmids and siRNAs

Full-length of GGTase-Iβ coding sequence was amplified from cDNA of CAL27 cells (human cancer cell line) with a high-fidelity DNA polymerase (TOYOBO, Osaka, Japan) by using standard polymerase chain reaction (PCR) techniques. The amplified gene was cloned into a pZeroBack/blunt vector (Tiangen, Beijing, China) and re-cloned into pEGFP-C1 plasmid at KpnI and XmaI sites. The constructs were confirmed by DNA sequencing. siRNAs for HDAC1, HDAC10, and HDAC11 was custom synthesized as described before [38,55]. Human GGTase-Iβ siRNA was synthesized in the following sequence: GGTase-Iβ-1 5′-GGCCUCUCAUGCUUAGUUATT-3′; GGTase-Iβ-2 5′-GGAGGAAAGUGGAAUUUGUTT-3′. siRNAs and plasmid were transfected using Lipofectamine 2000 (Invitrogen, Carlsbad, CA, USA) according to the manufacturer’s instructions.

### 4.4. Stable Transfection with Lentivirus

The process of lentivirus packaging was performed as previously described [38]. Briefly, the plasmid of pLVX-AcGFP-N1-RhoA Q63L was co-transfected into 293T cells with the packaging plasmids. Lentiviral supernatants were collected 48 h after transfection and then centrifuged (1000× *g* for 10 min at 4 °C). The supernatant was added to SACC-83 cells, which were subsequently cultured for 48 h. The infected cells were selected by 1 μg/mL puromycin for 10 days, and overexpression of RhoA Q63L mutant (constitutively active RhoA) in cells was confirmed by Western blot assay.

### 4.5. Real-Time Quantitative PCR

Total RNA was extracted using TRIzol reagent (Invitrogen). Reverse transcription and real-time PCR were performed as described previously [37]. The primers for human GGTase-Iβ are as follows: 5′-GCTGGATTTCTTACGGGATCG-3′ (sense) and 5′-CAGCCCGGAGAGTGCAAAA-3′ (anti-sense). The primers for human β-actin are as follows: 5′-CGGGAAATCGTGCGTGAC-3′ (sense) and 5′-CAGGCAGCTCGTAGCTCTT-3′ (anti-sense). All the primers were designed using the Primer Premier version 5.0 software (Premier, Canada). The efficiency of all the primers was confirmed by sequencing their conventional PCR products. Real-time PCR was performed using a 7500 real-time PCR system of Applied Biosystems (Invitrogen, Carlsbad, CA, USA). with FastStart Universal SYBR Green Master (Roche, Switzerland).

### 4.6. Protein Extraction and Western Blot Analysis

Whole-cell lysates were extracted using RIPA lysis buffer (Applygen, Beijing, China). Protein concentrations were determined by BCA protein assay (Thermo Scientific, Wilmington, DE, USA). Equal amounts of samples were subjected to 8% sodium dodecyl sulphate–polyacrylamide gel electrophoresis and transferred to a nitrocellulose filter membrane (Millipore, Billerica, MA, USA). The membrane was blocked with 5% fat-free milk in TBS-T (50 mmol/L Tris, pH 7.5; 150 mmol/L NaCl; 0.05% Tween 20) for 1 h. After incubation with primary antibodies diluted at 1:1000 in TBS-T containing 1% milk overnight at 4 °C, the membrane was washed extensively with TBS-T and then incubated with a secondary antibody conjugated with fluorophore diluted at 1: 10,000 for 1 h at room temperature. After extensive washing with TBS-T, the membrane was visualized using the Odyssey infrared imaging system (Odyssey LI-COR, Lincoln, NE, USA). For internal controls of equal loading, the blots were stripped in a stripping buffer (100 mmol/L 2-mercaptoethanol; 2% sodium dodecyl sulphate; 62.5 mmol/L Tris, pH 6.8) and re-probed with β-actin antibody.

### 4.7. Cell Proliferation Assay

Cell proliferation assay was performed using Cell Counting Kit-8 (CCK-8, Dojindo, Kumamoto, Japan) according to the manufacturer’s instructions. Briefly, the cells were seeded onto 96-well plates (1.5 × 10^3^ cells per well) and treated with different reagents. After 48 h treatment, 10 μL of CCK-8 were added to each well containing 100 μL growth medium. After incubation at 37 °C for 3 h, absorbance at 450 nm was determined.

### 4.8. Assessment of Cell Apoptosis

Cells were washed with phosphate-buffered saline (PBS) thrice, fixed with 4% paraformaldehyde for 5 min, and incubated with 5 μg/mL 4,6-diamidino-2-phenylindole dihydrochloride (DAPI) in the dark for 3 min at room temperature. After being washed with PBS, the cells were examined under a fluorescence microscope (Nikon, Japan). Cells presenting features of nuclear condensation and fragmentation were identified as apoptotic cells and counted within the six randomly selected fields. The rate of apoptotic cells was presented as mean ± SD of at least three independent experiments.

### 4.9. Transwell Migration and Invasion Assay

Cell migration and invasion assays were performed in transwell chambers (Corning Costar, Corning, NY, USA) by using a polycarbonate membrane as described previously [55]. Briefly, for migration assays, the cells were seeded at 1 × 10^5^ cells per well in serum-free medium in the upper chambers; the lower chambers contained the growth medium with 10% fetal bovine serum. The cells were then incubated with the reagents in the lower chambers for 16 h. Cells on the top surface of the membrane were wiped off, whereas those on the bottom surface were fixed with 4% paraformaldehyde and stained with 0.01% crystal violet, and examined under a light microscope, counted, and averaged by the number of six randomly selected fields. The same procedure was performed for transwell invasion assay, except that the upper chambers were coated with 20 μg of extracellular matrix gel prior to the seeding of the cells (BD Bioscience, San Jose, CA, USA).

### 4.10. Xenograft Tumor Inoculation

All animal experiments were performed in compliance with the National Institutes of Health guidelines for the care and use of laboratory animals. Nude mice (NU/NU, five weeks old) were purchased from Beijing Vital River Laboratory Animal Technology Co., Ltd. (Beijing, China). The care and treatment of experimental animals followed the institutional guidelines. The experimental protocols were approved by the Peking University Biomedical Ethics Committee Experimental Animal Welfare Ethics Branch (NO. LA2008-004, 21 November, 2008). Mice were randomly allocated to each group (*n* = 5). CAL27 cells were subcutaneously inoculated (1 × 10^6^ cells/mouse) in the right flanks of mice (*n* = 5). After 10 days, mice from each group received either CI994 (20 mg/kg/2 days, dissolved in 0.5% carboxy-methyl cellulose), or atorvastatin (10 mg/kg/day, dissolved in 0.5% carboxy-methyl cellulose), or both by lavage for three weeks, whereas the mice that only received 0.5% carboxy-methyl cellulose by lavage were defined as the control group. The mice were killed, and the weights of xenograft tumors were measured.

### 4.11. Luciferase Assay

Luciferase assay was performed as described previously [55]. Briefly, GGTase-Iβ reporter plasmid (containing promoter of GGTase-Iβ range from −1141/+36; transcriptional start site was defined as +1) was transfected into SACC-83 cells in a 12-well plate. The transfected cells were lysed in a cell lysis buffer 36 h after transfection. Luciferase activity was measured with a LB960 microplate luminometer (Berthold, Berlin, Germany) using luciferin as the substrate, according to the manufacturer’s instructions (Promega, Fitchburg, WI, USA).

### 4.12. Statistical Analysis

Statistical analysis was performed using SPSS 20 for Windows. All experiments were repeated three times, and all data were presented as mean ± standard deviation (SD). Differences between multiple groups were analyzed by one-way analysis of variance (ANOVA). *P* < 0.05 was considered to indicate statistical significance.

## 5. Conclusions

Inhibiting HDAC1 could enhance the anti-cancer effects of statins through downregulation of GGTase-Iβ expression both in vitro and in vivo, without severe damage to non-tumor cells. RhoA was determined as the key downstream effector of the combination of statin and HDAC1 inhibitor. The combination of HDAC1 inhibitor and statin would be a potential new regimen for cancer therapeutics.

## Supplementary Materials

Supplementary materials can be found at www.mdpi.com/1422-0067/18/5/1010/s1.
