# Supplementary material for: Inhibiting HDAC1 Enhances the Anti-Cancer Effects of Statins through Downregulation of GGTase-Iβ Expression
Source: Int J Mol Sci. 2017 May 8;18(5):1010. doi: 10.3390/ijms18051010 (PMC5454923; doi:10.3390/ijms18051010)
Supplement: Supplementary file 1 [file ijms-18-01010-s001.pdf]

Inhibiting HDAC1 Enhances the Anti-Cancer Effects of Statins through Downregulation of GGTase-Iβ Expression

Ran Li and Ye-Hua Gan

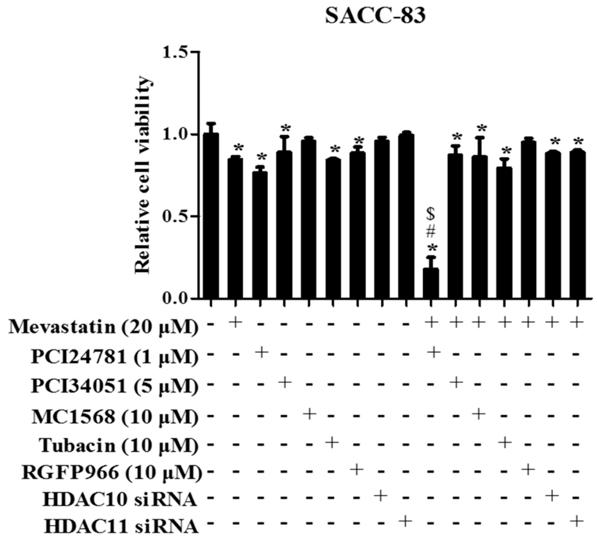

Figure S1. HDAC1 & 2 may be involved in the enhancement of mevastatin-induced cell proliferation inhibition by pan-HDAC inhibitor. SACC-83 cells were exposed to various kinds of HDAC inhibitors or siRNAs (PCI24781, inhibitor of HDAC1, 2, 3, 6, 8, 10; PCI34051, inhibitor of HDAC3; MC1568, inhibitor of HDAC4, 5, 7, 9; tubacin, inhibitor of HDAC6; RGFP966, inhibitor of HDAC8; siRNA of HDAC10 or 11), or together with mevastatin for 48 h. Cell viability was assessed by CCK8 assay. *$P <$ 0.05 vs. the control group; # P <0.05 vs. mevastatin group; \$ $P < 0.05$ vs. PCI24781 group, $n = 4$.

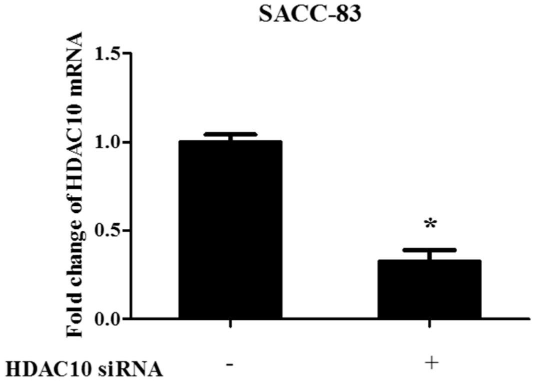

Figure S2. Confirmation of HDAC10 knockdown efficiency**.** siRNA of HDAC10 was transfected to SACC-83 cells. After 48 h treatment, total RNA was extracted and the mRNA expression of HDAC10 was assessed by real-time PRC. * $P < 0.05$, vs. control group, $n = 3$.

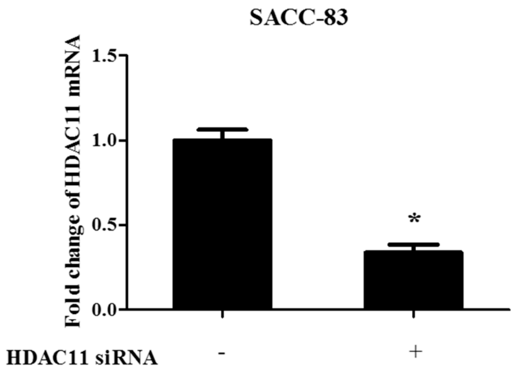

Figure S3. Confirmation of HDAC11 knockdown efficiency. siRNA of HDAC11 was transfected to SACC-83 cells. After 48 h treatment, total RNA was extracted and the mRNA expression of HDAC11 was assessed by real-time PRC. * $P < 0.05$, vs. control group, $n = 3$.

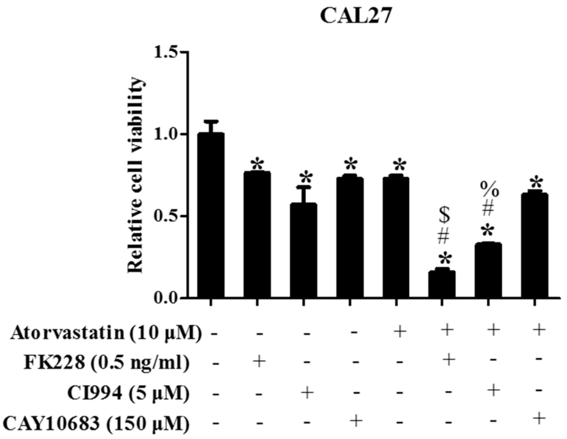

Figure S4. Inhibiting HDAC1, but not HDAC2, enhanced the atorvastatin-induced inhibition of cell proliferation in CAL-27 cells. CAL-27 cells were either exposed to various kinds of HDAC inhibitors (FK228, inhibitor for HDAC1&2; CI994, inhibitor for HDAC1; CAY10683, inhibitor for HDAC2), or along with atorvastatin for 48 h. Cell viability was assessed by a CCK8 assay. * $P < 0.05$ vs. the control group; # $P < 0.05$ vs. atorvastatin group; $ $P < 0.05$ vs. FK228 group; % $P < 0.05$ vs. CI994 group, $n = 4$.

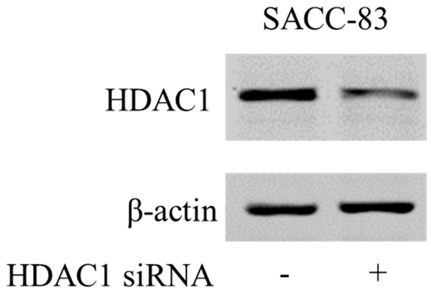

Figure S5. Confirmation of HDAC1 knockdown efficiency. SACC-83 cells were transfected with scrambled or HDAC1 siRNA; after 30 h treatment, total protein was extracted and subjected to Western blot for analysis.

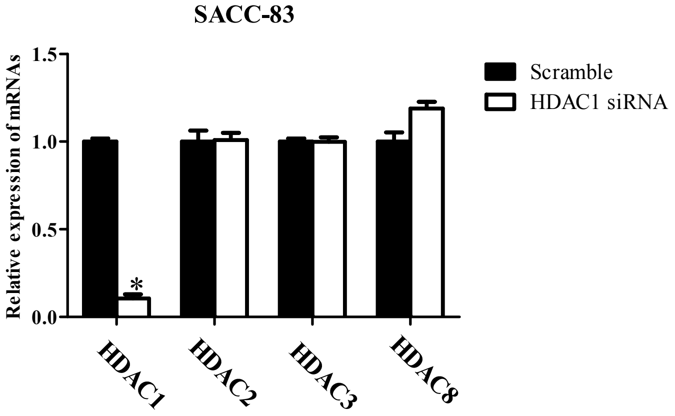

Figure S6. Confirmation of HDAC1 siRNA specificity. Scrambled and HDAC1 siRNAs were transfected to SACC-83 cells for 30 h, then total RNA was extracted and subjected to real-time PCR. * $P < 0.05$. $n = 3$.

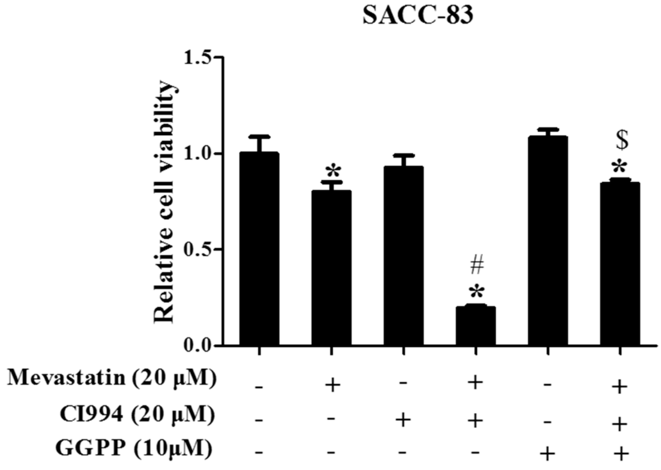

Figure S7. GGPP abolished the enhancement of mevastatin-induced inhibition of cell viability by CI994. SACC-83 cells were exposed to different kinds of treatments for 48 h, and then cell viability was assessed by a CCK8 assay. * $P < 0.05$ vs. the control group; # $P < 0.05$ vs. atorvastatin or CI994 group; $\$$ $P < 0.05$ vs. the group of combinational treatment with mevastatin and CI994, $n = 4$.

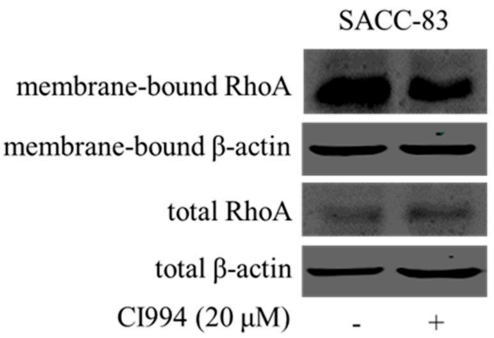

Figure S8. CI994 (HDAC1 inhibitor) inhibited membrane-translocation (activation) of RhoA. SACC-83 cells were exposed to CI994 (20 μM) for 30 h, and then membrane and total proteins were extracted and subjected to Western blot for analysis.

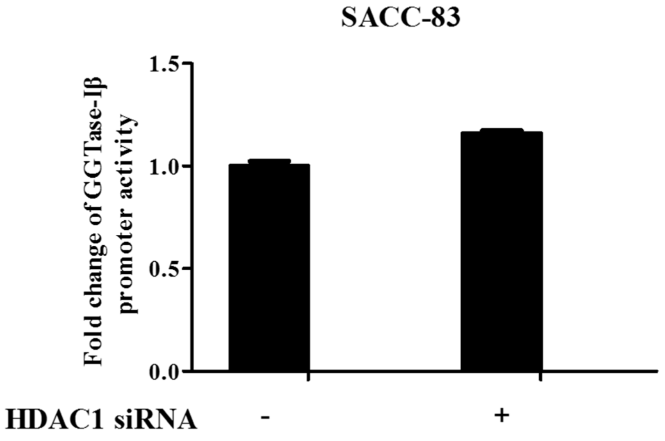

Figure S9. Inhibiting HDAC1 did not influence the promoter activity of GGTase-Iβ. Scrambled and HDAC1 siRNA were transfected to SACC-83 cells. After 12 h transfection, pGL3-enhancer vector containing GGTase-Iβ promoter region was transfected to the cells, and incubated for another 36 h. Total protein was extracted and subjected to luciferase assay for analysis, *n* = 4.

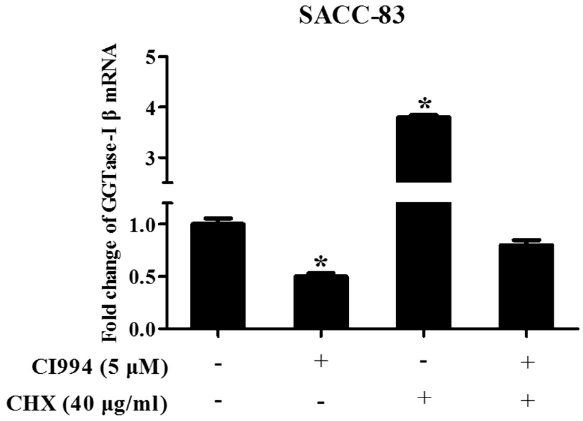

Figure S10. Inhibition of new protein synthesis by CHX rescued CI994-induced downregulation of GGTase-Iβ mRNA. SACC-83 cells were exposed to either CI994, or cycloheximide (CHX), or both for 30 h. mRNA expression of GGTase-Iβ was quantified by real-time PCR. * $P < 0.05$ vs. the control group, $n = 3$.

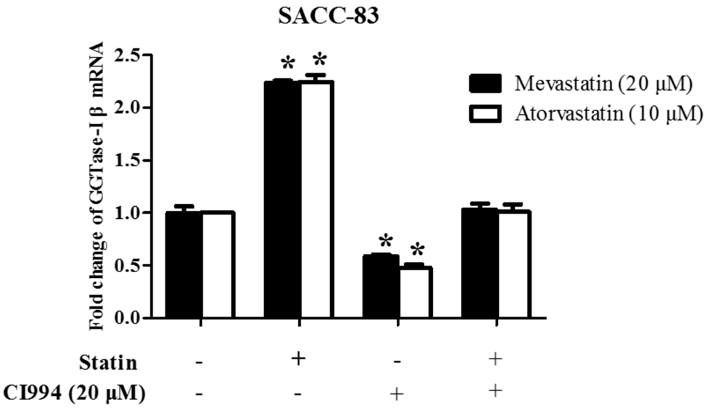

Figure S11. CI994 blocked statin-induced upregulation of GGTase-Iβ mRNA. SACC-83 cells were treated with statin (mevastatin 20 μM/atorvastatin 10 μM), or CI994 (20 μM), or both for 30 h. The mRNA expression of GGTase-Iβ was quantified by real-time PCR. *$P < 0.05$ vs. the control group, $n = 3$.

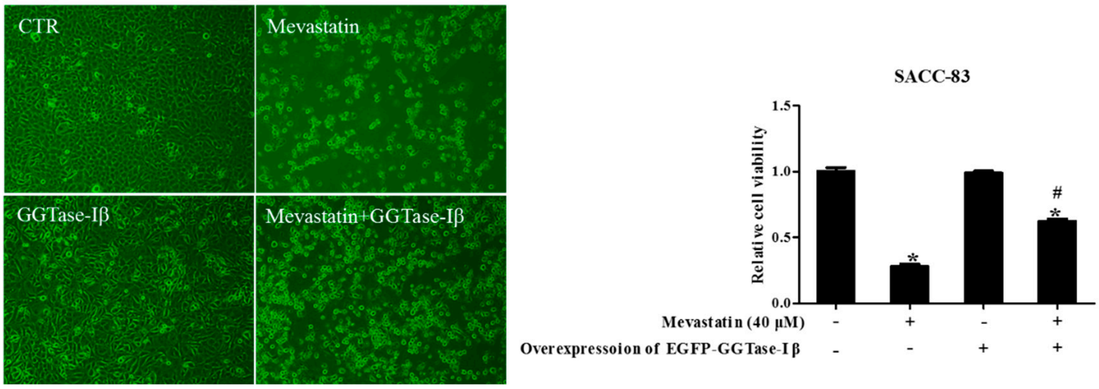

Figure S12. Overexpression of GGTase-Iβ partially rescued mevastatin-induced inhibition of cell viability. Microscopy photographs and cell viability of SACC-83 cells. SACC-83 cells were transfected with either EGFP fused GGTase-Iβ, or an empty vector (pEGFP-C1); after 12 h transfection, cells were exposed to mevastatin at a concentration of 40 μM for another 48 h. * $P < 0.05$ vs. the control group; # $P < 0.05$ vs. mevastatin group.

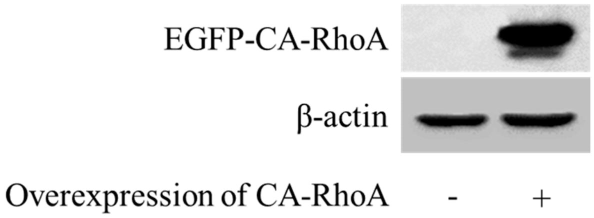

Figure S13. Confirmation of CA-RhoA overexpression. SACC-83 cells were either stably transfected with EGFP-fused constitutively active RhoA or not. Total cell lysate was collected and subject to analysis by Western blot assay.
